# Supplementary material for: Breaking Dynamic Behavior in 3D Covalent Organic Framework with Pre-Locked Linker Strategy
Source: Nanomaterials (Basel). 2024 Feb 7;14(4):329. doi: 10.3390/nano14040329 (PMC10891907; doi:10.3390/nano14040329)
Supplement: Supplementary file 1 [file nanomaterials-14-00329-s001.zip › nanomaterials-2818660-supplementary.pdf]

nanomaterials

Breaking Dynamic Behavior in 3D Covalent Organic Framework with Pre-Locked Linker Strategy

Xiaohong Chen [1,†], Chengyang Yu [2,†], Yusran Yusran [1,*], Shilun Qiu [1] and Qianrong Fang [1,*]

[1] College of Chemistry, State Key Laboratory of Inorganic Synthesis and Preparative Chemistry, Jilin University, Changchun 130012, China; cxh22@mails.jlu.edu.cn (X.C.); sqiu@jlu.edu.cn (S.Q.)
[2] College of Chemistry and Environmental Engineering, Wuhan Polytechnic University, Wuhan 430023, China; 22113097@whpu.edu.cn
* Corresponding authors: postys@jlu.edu.cn (Y.Y.); qrfang@jlu.edu.cn (Q.F.)
† These authors contributed equally.

Supporting Information

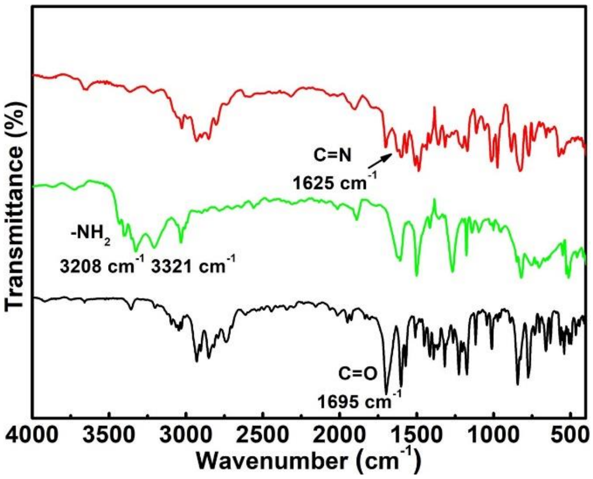

Figure S1. FT-IR spectra of TFPA (black), BD (green) and JUC-594 (red).

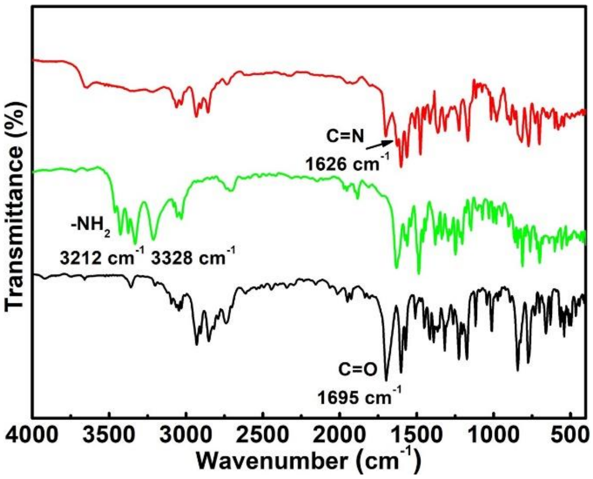

Figure S2. FT-IR spectra of TFPA (black), DPP (green) and JUC-595 (red).

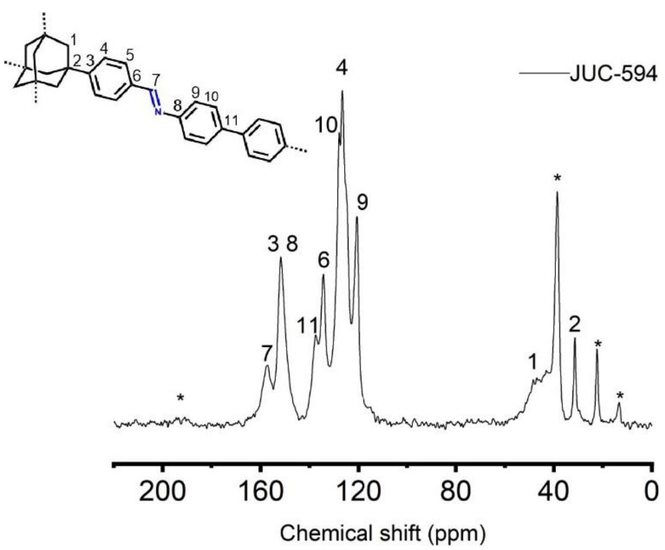

Figure S3. The ss ^{13}C CP/MAS NMR spectrum of JUC-594.

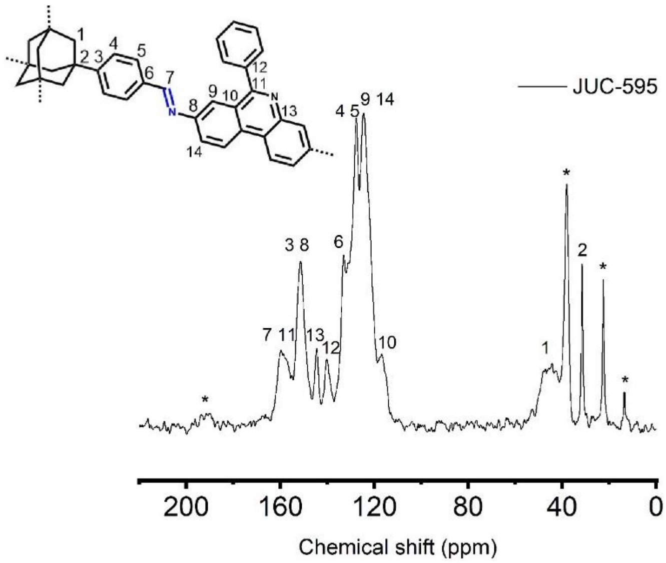

Figure S4. The ss ^{13}C CP/MAS NMR spectrum of JUC-595.

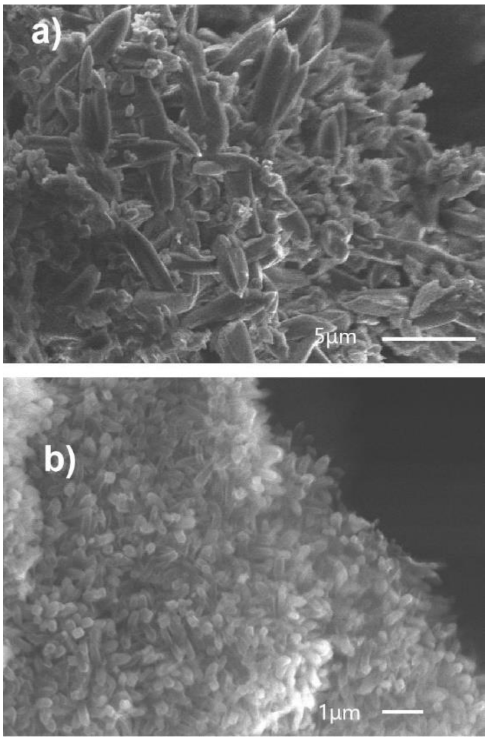

Figure S5. SEM images of JUC-594 (a) and JUC-595 (b).

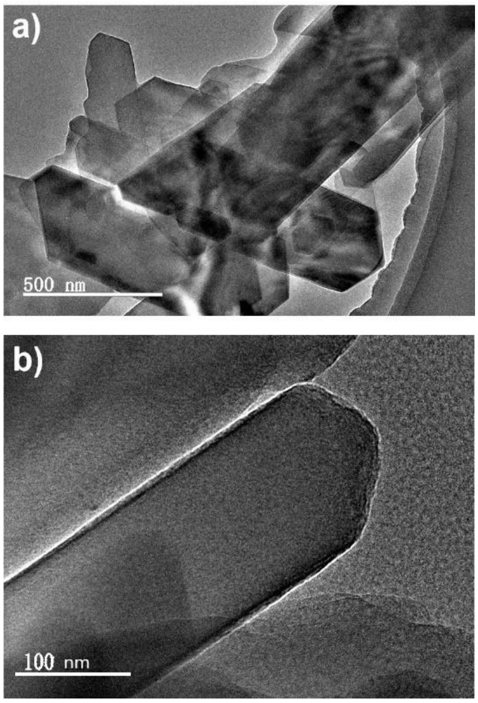

Figure S6. TEM images of a) JUC-594 and b) JUC-595.

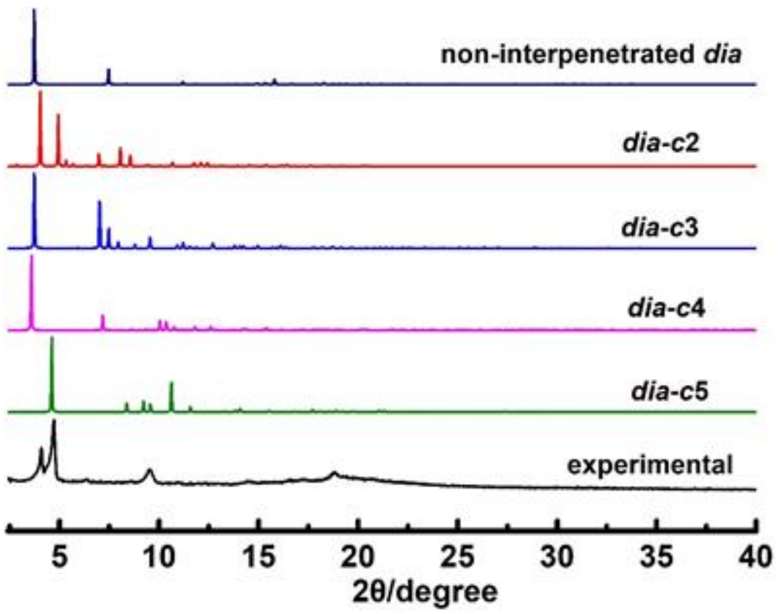

Figure S7. PXRD profiles JUC-594 experimental and simulated structures calculated from the non-, 2-, 3-, 4-, and 5-fold interpenetrated **dia** nets.

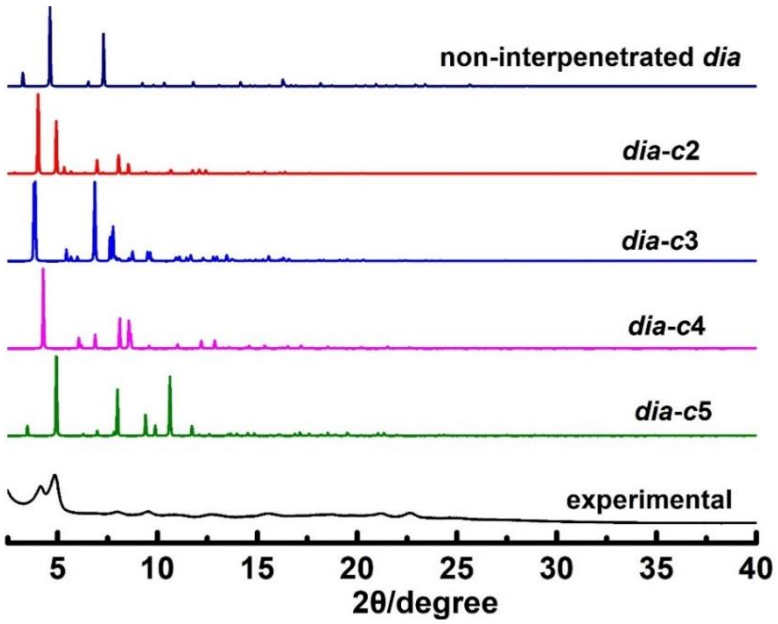

Figure S8. PXRD profiles JUC-595 experimental and simulated structures calculated from the non-, 2-, 3-, 4-, and 5-fold interpenetrated **dia** nets.

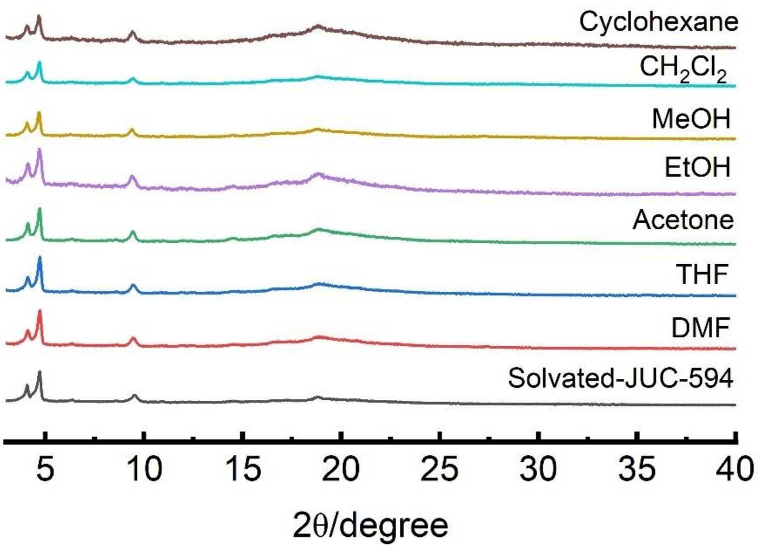

Figure S9. The overlay PXRD pattern of JUC-594 after immersing into various organic solvents for 24 h.

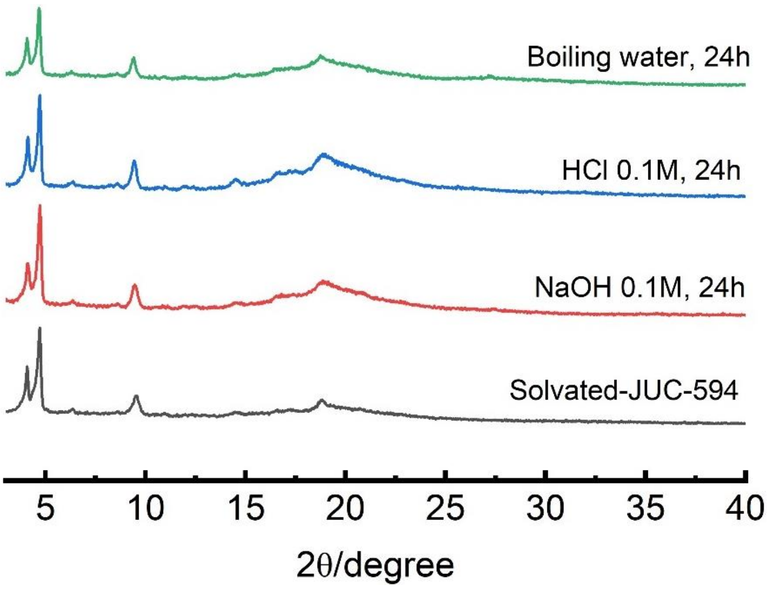

Figure S10. The overlay PXRD pattern of JUC-594 after immersing into boiling water, HCl 0.1 M, and NaOH 0.1 M for 24 h.

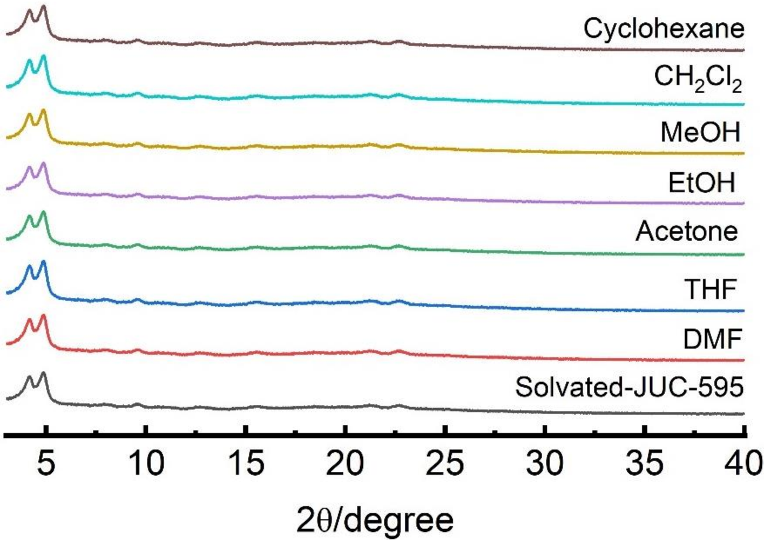

Figure S11. The overlay PXRD pattern of JUC-595 after immersing into various organic solvents for 24 h.

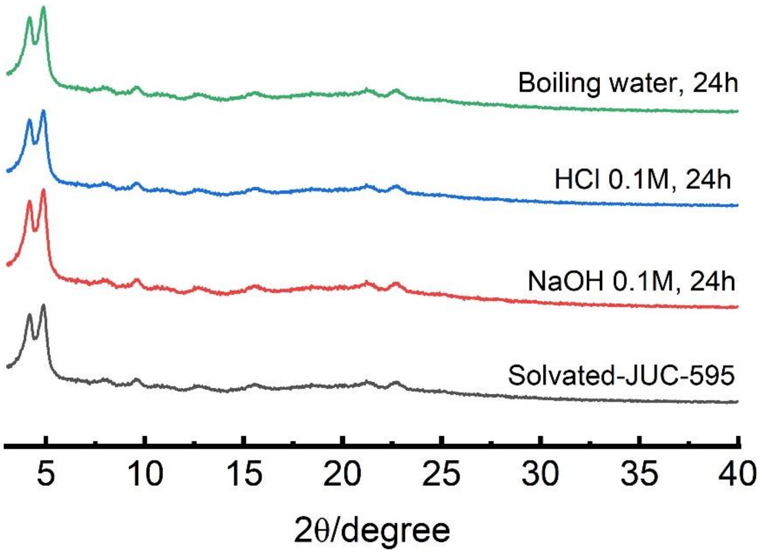

Figure S12. The overlay PXRD pattern of JUC-595 after immersing into boiling water, HCl 0.1 M, and NaOH 0.1 M for 24 h.

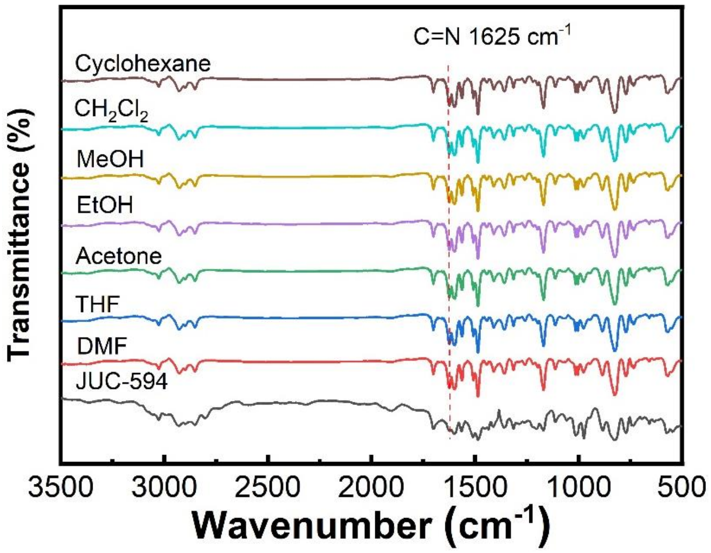

Figure S13. The overlay FT-IR spectra of JUC-594 after immersing into organic solvents for 24 h.

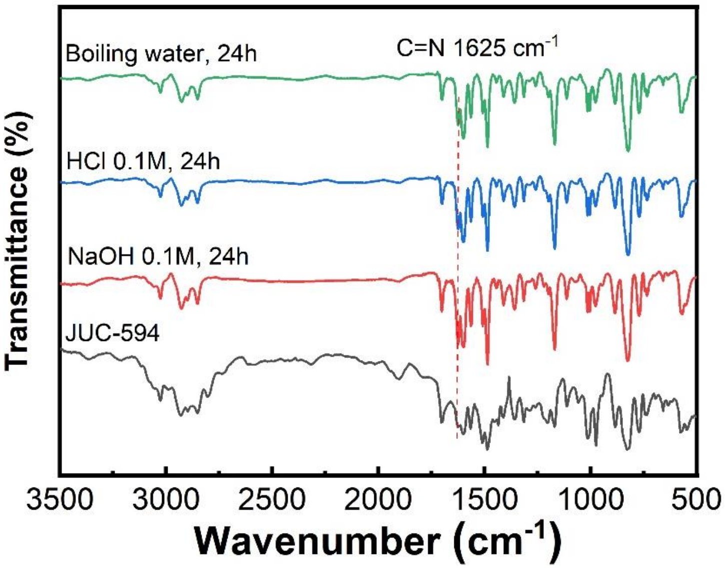

Figure S14. The overlay FT-IR spectra of JUC-594 after immersing into boiling water, HCl 0.1 M, and NaOH 0.1 M for 24 h.

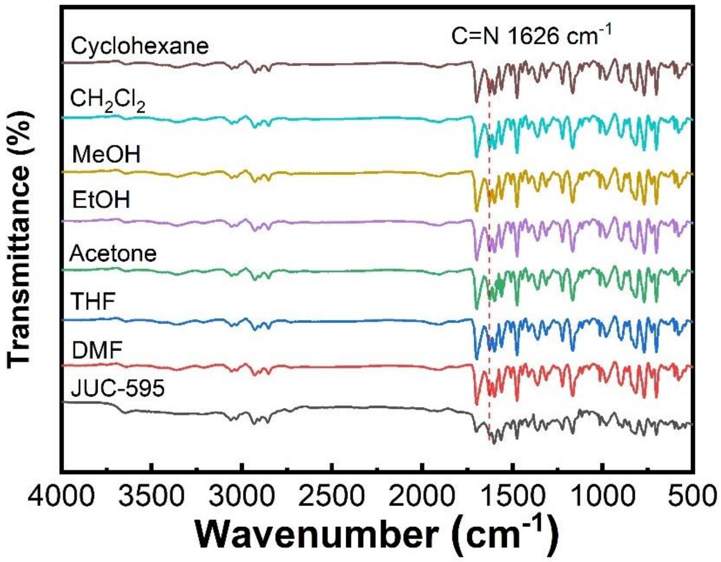

Figure S15. The overlay FT-IR spectra of JUC-595 after immersing into organic solvents, for 24 h.

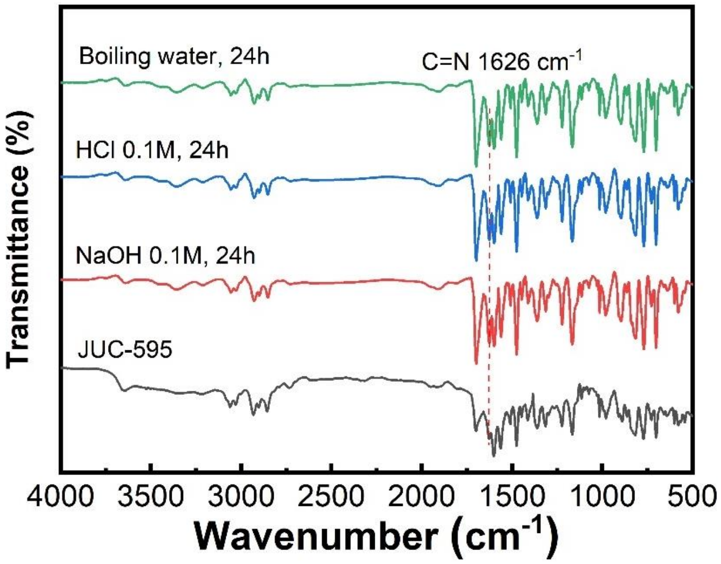

Figure S16. The overlay FT-IR spectra of JUC-595 after immersing into boiling water, HCl 0.1 M, and NaOH 0.1 M for 24 h.

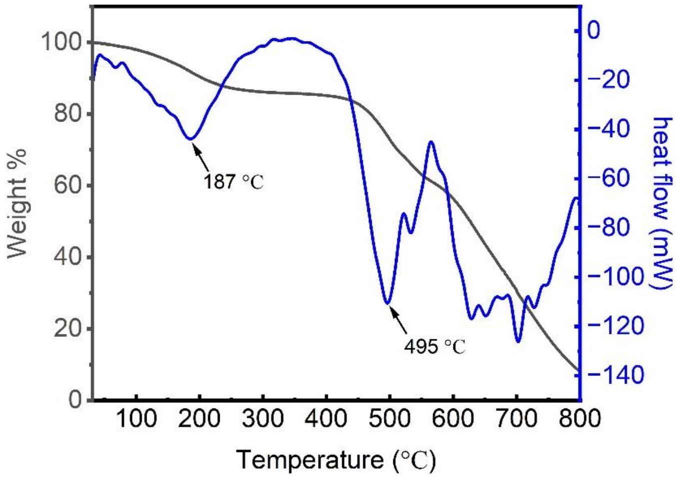

Figure S17. TGA-DSC curve of JUC-594.

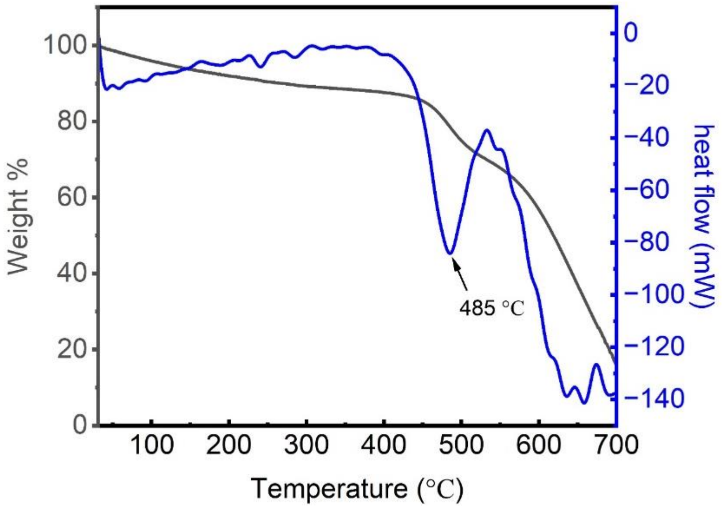

Figure S18. TGA-DSC curve of JUC-595.

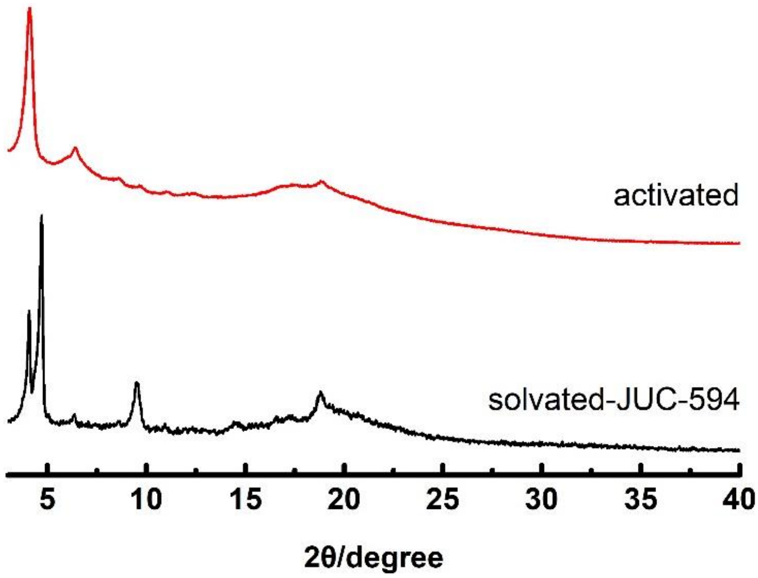

Figure S19. PXRD profiles JUC-594 upon solvated and activated.

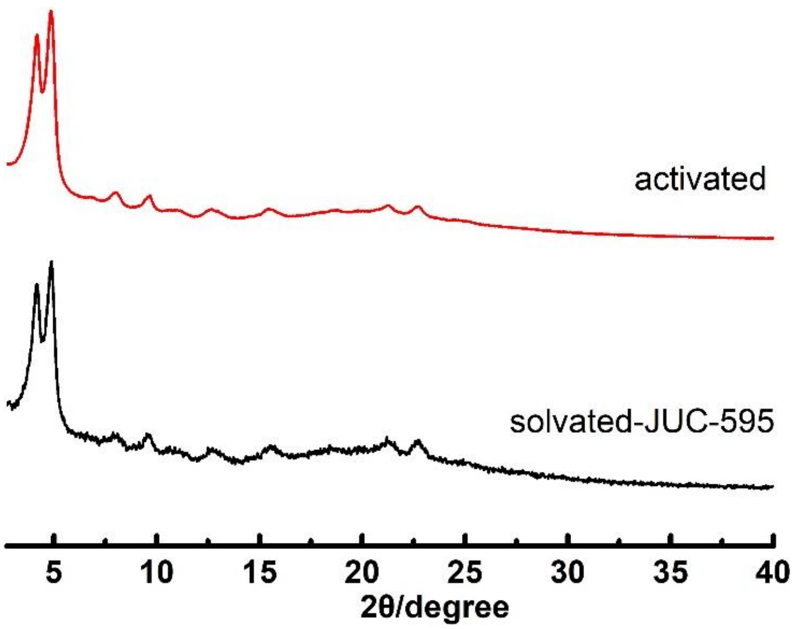

Figure S20. PXRD profiles JUC-595 upon solvated and activated.

Section S5. Porosity analysis

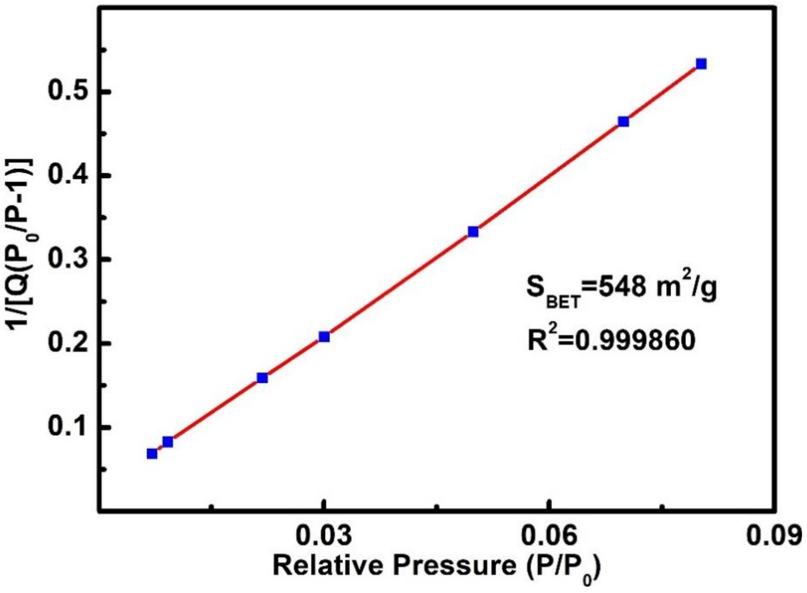

Figure S21. BET plot of JUC-594 calculated from N_2 adsorption isotherm at 77 K.

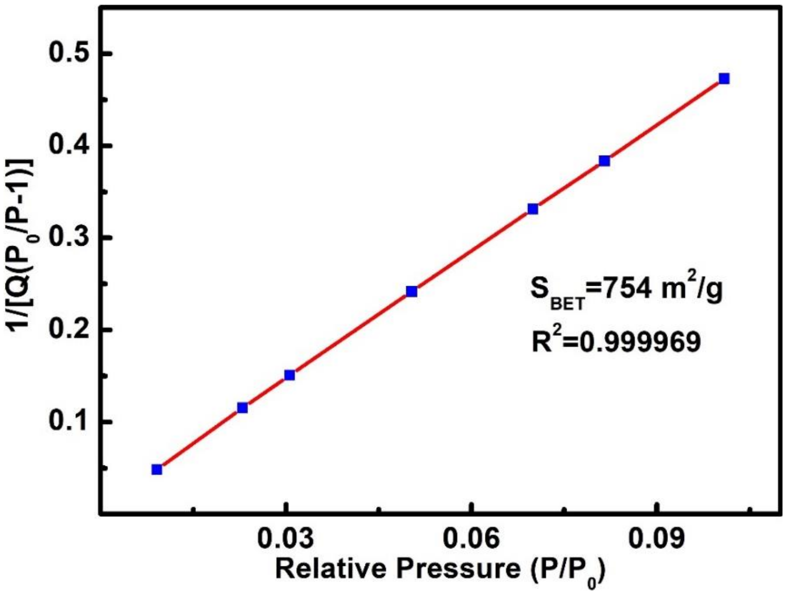

Figure S22. BET plot of JUC-595 calculated from N_2 adsorption isotherm at 77 K.

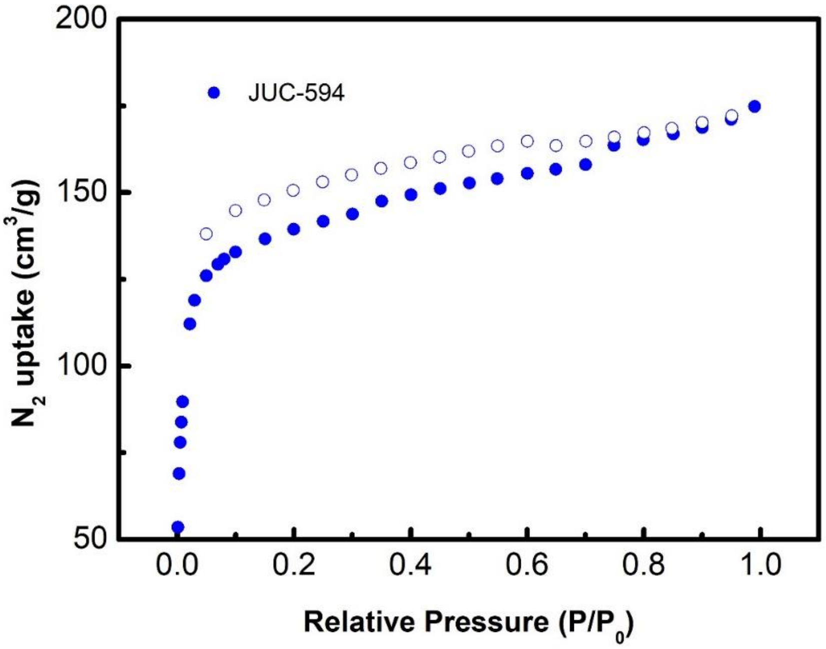

Figure S23. N_2 adsorption isotherm of JUC-594 at 77 K.

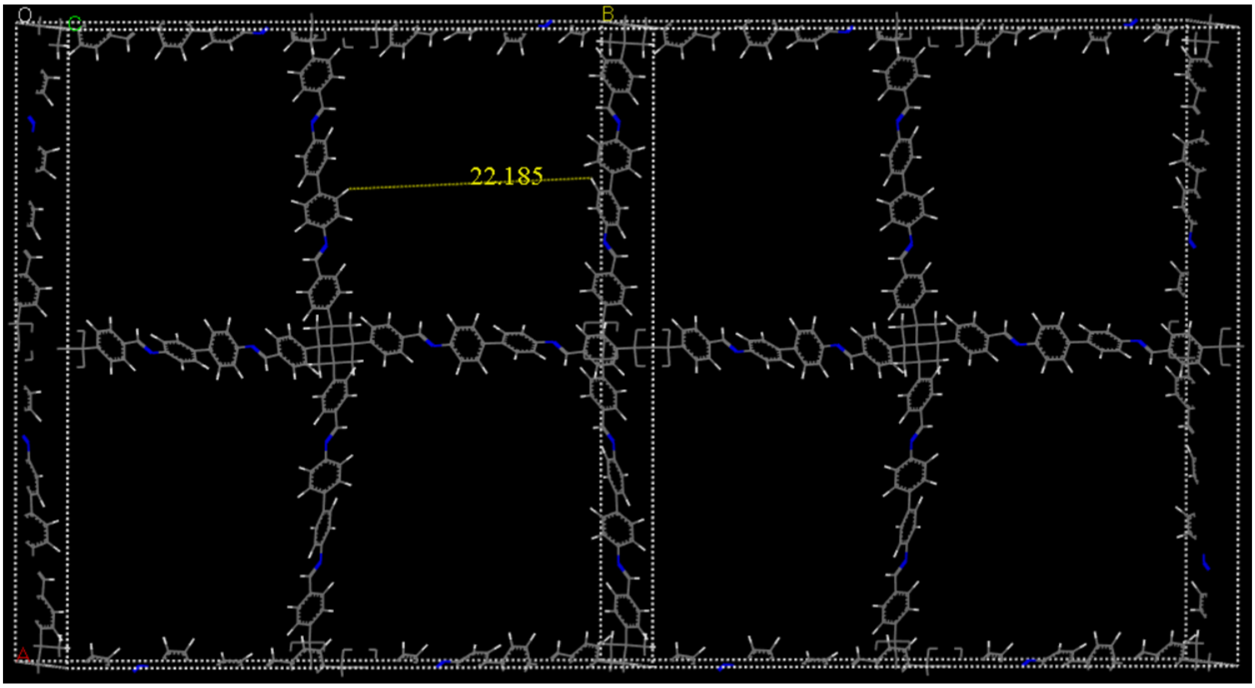

Figure S24. Pore size of simulated non-contracted JUC-594 (2.2 nm).

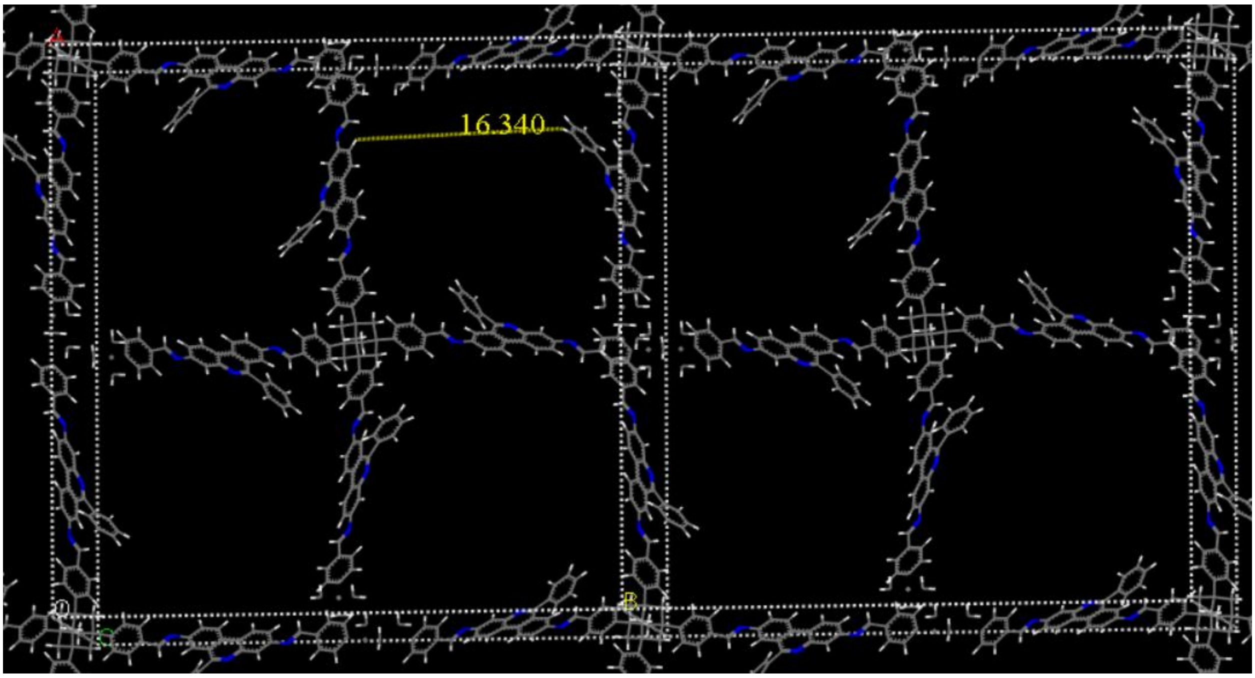

Figure S25. Pore size of simulated non-contracted JUC-595 (1.6 nm).

Table S1. Unit cell parameters and fractional atomic coordinates for JUC-594 calculated based on the 2-fold interpenetrated **dia** net.

Space group		P-4B2	
Calculated unit cell		a = b = 42.2408 Å, c = 31.5544 Å, $\alpha = \beta = \gamma = 90°$	
Measured unit cell		a = b = 42.2149 Å, c = 31.5409 Å, $\alpha = \beta = \gamma = 90°$	
Pawley refinement		R_p = 3.81%, R_{wp} = 5.05%	
Atoms	X	Y	Z
C1	0.4997	0.9100	0.9668
C2	0.4995	0.8816	0.9441

C3	0.4997	0.8817	0.8997
C4	0.5001	0.9109	0.8783
C5	0.5003	0.9393	0.9009
C6	0.5000	0.9394	0.9456
C7	0.4994	0.8514	0.8765
N8	0.5006	0.8507	0.8354
C9	0.5098	0.8254	0.7670
C10	0.5095	0.7991	0.7402
C11	0.4991	0.7695	0.7551
C12	0.4888	0.7669	0.7973
C13	0.4892	0.7933	0.8242
C14	0.5000	0.8228	0.8093
C15	0.4734	0.7201	0.7274
C16	0.4733	0.6935	0.7011
C17	0.4989	0.6877	0.6735
C18	0.5241	0.7092	0.6725
C19	0.5241	0.7358	0.6988
C20	0.4989	0.7415	0.7267
C21	0.4816	0.6362	0.6462
N22	0.5003	0.6606	0.6459
C23	0.4657	0.5838	0.6173
C24	0.4697	0.5586	0.5892
C25	0.4942	0.5587	0.5588
C26	0.5145	0.5852	0.5575
C27	0.5105	0.6105	0.5857
C28	0.4861	0.6100	0.6159
C29	0.5023	0.4698	0.5288
C30	0.4681	0.5276	0.4999
C31	-0.0303	0.5000	1.0283
H32	0.4995	0.9091	1.0011
H33	0.4992	0.8594	0.9613
H34	0.5003	0.9117	0.8440
H35	0.5005	0.9612	0.8830
H36	0.4989	0.8297	0.8947
H37	0.5177	0.8481	0.7549
H38	0.5171	0.8020	0.7077
H39	0.4808	0.7445	0.8098
H40	0.4805	0.7904	0.8562
H41	0.4533	0.7242	0.7480
H42	0.4529	0.6781	0.7022
H43	0.5439	0.7052	0.6514
H44	0.5442	0.7517	0.6978
H45	0.4628	0.6341	0.6693
H46	0.4468	0.5828	0.6404
H47	0.4536	0.5387	0.5916
H48	0.5338	0.5869	0.5352
H49	0.5266	0.6304	0.5838
H50	0.4664	0.5493	0.4799
H51	0.4465	0.5262	0.5199

C52	0.5000	0.5000	0.5560
C53	0.9704	0.5297	1.0000
C54	0.9703	0.4703	1.0000
C55	1.0000	0.5000	0.9442

Table S2. Unit cell parameters and fractional atomic coordinates for JUC-595 calculated based on the 2-fold interpenetrated **dia** net.

Space group		P-4B2	
Calculated unit cell		a = b = 44.7519 Å, c = 27.9163 Å, $\alpha = \beta = \gamma = 90°$	
Measured unit cell		a = b = 47.5409 Å, c = 29.6538 Å, $\alpha = \beta = \gamma = 90°$	
Pawley refinement		R_p = 0.91%, R_{wp} = 1.37%	
Atoms	X	Y	Z
C1	0.4741	0.9401	0.9027
C2	0.4720	0.9152	0.8726
C3	0.4940	0.8931	0.8732
C4	0.5183	0.8962	0.9045
C5	0.5206	0.9211	0.9346
C6	0.4984	0.9436	0.9344
N7	0.5101	0.8454	0.8415
C8	0.4909	0.8671	0.8410
C9	0.5247	0.7938	0.8252
C10	0.5239	0.7678	0.7972
C11	0.5077	0.7670	0.7538
C12	0.4928	0.7934	0.7392
C13	0.4936	0.8192	0.7674
C14	0.5091	0.8194	0.8112
C15	0.4905	0.7367	0.6844
C16	0.4896	0.7095	0.6600
C17	0.5050	0.6847	0.6772
C18	0.5212	0.6871	0.7200
C19	0.5230	0.7146	0.7448
C20	0.5070	0.7398	0.7273
C21	0.5198	0.6340	0.6544
N22	0.5025	0.6572	0.6508
C23	0.5347	0.5835	0.6259
C24	0.5305	0.5584	0.5969
C25	0.5059	0.5563	0.5654
C26	0.4854	0.5804	0.5649
C27	0.4897	0.6054	0.5938
C28	0.5146	0.6073	0.6244
N29	0.5392	0.7435	0.8123
C30	0.5397	0.7173	0.7877
C31	0.4976	0.4715	0.5329
C32	0.5003	0.0286	0.9672
C33	0.5258	0.4698	0.5002
C34	0.4286	0.3398	0.8736
C35	0.4465	0.3172	0.8544
C36	0.4417	0.3071	0.8074
C37	0.4181	0.3193	0.7805

C38	0.4002	0.3419	0.7998
C39	0.4055	0.3522	0.8463
H40	0.4564	0.9565	0.9009
H41	0.4529	0.9134	0.8488
H42	0.5357	0.8794	0.9056
H43	0.5399	0.9222	0.9578
H44	0.4716	0.8662	0.8178
H45	0.5371	0.7940	0.8585
H46	0.4805	0.7950	0.7061
H47	0.4824	0.8389	0.7542
H48	0.4778	0.7546	0.6688
H49	0.4767	0.7078	0.6273
H50	0.5316	0.6674	0.7348
H51	0.5390	0.6342	0.6779
H52	0.5540	0.5843	0.6493
H53	0.5469	0.5406	0.5992
H54	0.4659	0.5804	0.5422
H55	0.4736	0.6235	0.5920
H56	0.5463	0.4682	0.5227
H57	0.5244	0.4493	0.4780
H58	0.4325	0.3477	0.9097
H59	0.4643	0.3078	0.8759
H60	0.4136	0.3112	0.7446
H61	0.3822	0.3513	0.7788
H62	0.3916	0.3696	0.8613
H63	0.5198	0.4977	0.5880
H64	-0.0009	0.4801	0.0879
H65	0.9716	0.5491	0.0216
H66	0.9517	0.4719	-0.0230
C67	0.5000	0.5000	0.5642
C68	0.0000	0.5000	0.0641
C69	0.9717	0.5283	0.0000
C70	0.9721	0.4721	0.0000
